# Modulation of μ‐opioid receptor activation by acidic pH is dependent on ligand structure and an ionizable amino acid residue

**DOI:** 10.1111/bph.14810

**Published:** 2019-12-05

**Authors:** Johanna Meyer, Giovanna Del Vecchio, Viola Seitz, Nicolas Massaly, Christoph Stein

**Affiliations:** ^1^ Department of Experimental Anesthesiology, Campus Benjamin Franklin Charité—Universitätsmedizin Berlin Germany

## Abstract

**Background and Purpose:**

Adverse side effects of conventional opioids can be avoided if ligands selectively activate peripheral opioid receptors in injured tissue. Injury and inflammation are typically accompanied by acidification. In this study, we examined influences of low pH and mutation of the ionizable amino acid residue H297^6.52^ on μ‐opioid receptor binding and signalling induced by the μ‐opioid receptor ligands fentanyl, DAMGO, and naloxone.

**Experimental Approach:**

HEK 293 cells stably transfected with μ‐opioid receptors were used to study opioid ligand binding, [^35^S]‐GTPγS binding, and cAMP reduction at physiological and acidic pH. We used μ‐opioid receptors mutated at H297^6.52^ to A (MOR‐H297^6.52^A) to delineate ligand‐specific interactions with H297^6.52^.

**Key Results:**

Low pH and the mutant receptor MOR‐H297^6.52^A impaired naloxone binding and antagonism of cAMP reduction. In addition, DAMGO binding and G‐protein activation were decreased under these conditions. Fentanyl‐induced signalling was not influenced by pH and largely independent of H297^6.52^.

**Conclusions and Implications:**

Our investigations indicate that low pH selectively impairs μ‐opioid receptor signalling modulated by ligands capable of forming hydrogen bonds with H297^6.52^. We propose that protonation of H297^6.52^ at acidic pH reduces binding and subsequent signalling of such ligands. Novel agonists targeting opioid receptors in injured tissue might benefit from lack of hydrogen bond formation with H297^6.52^.

**What is already known**

- Selective activation of opioid receptors in peripheral injured tissue precludes adverse opioid side effects.
- Opioid receptor activation can be restricted to low pH environments in injured and inflamed tissue.

**What this study adds**

- The modulation of μ‐opioid receptor activation by pH is dependent on ligand structure.
- An ionizable amino acid residue (H297^6.52^) in μ‐opioid receptors is crucial for ligand binding and signalling.

**What is the clinical significance**

- Novel injury‐specific opioid agonists might benefit from lack of hydrogen bond formation with H297^6.52^.

**Abbreviations**
CTX: cholera toxin
DAMGO: [D‐Ala2,N‐Me‐Phe4,Gly5‐ol]‐enkephalin
EPPS: 4‐(2‐Hydroxyethyl)‐1‐piperazinepropanesulfonic acid
HEK MOR‐H297A: HEK293 cells stably transfected with μ‐opioid receptor mutant H297^6.52^A
HEK MOR‐WT: HEK293 cells stably transfected with μ‐opioid receptor wild type
MES: 2‐(N‐Morpholino)ethanesulfonic acid
NLX: naloxone
PTX: pertussis toxin
WT: wild type

## 1 INTRODUCTION

The https://www.guidetopharmacology.org/GRAC/ObjectDisplayForward?objectId=319 is the clinically most important member of the opioid receptor family of class A GPCRs. Opioid drugs are currently the most efficient treatment for the treatment of strong pain, yet they produce severe side effects, which limit their applicability and have contributed to the current opioid epidemic (World Drug Report, 2018). A promising approach to reduce side effects is to restrict the activity of opioids to peripheral sensory neurons in injured tissues (Tiwari et al., 2016; Vanderah et al., 2004). Peripheral opioid receptors contribute a significant proportion to the analgesic effect of systemically applied opioids (Jagla, Martus, & Stein, 2014), and opioid analgesia is enhanced in inflamed tissue (see Stein, 2018). Inflammation is, amongst other factors, characterized by low pH, and pH‐dependent modulation of receptor function has been reported for several GPCRs (Ghanouni et al., 2000; Lans, Dalton, & Giraldo, 2015; Ludwig et al., 2003; Pert & Snyder, 1973; Selley, Breivogel, & Childers, 1993; Spahn et al., 2017; Vetter, Kapitzke, Hermanussen, Monteith, & Cabot, 2006). Whereas we previously investigated a novel pH‐dependent opioid agonist (Spahn et al., 2017), we now concentrate on the receptor and examine whether protonation of a specific amino acid residue affects recognition of conventional ligands at acidic pH.

**Figure 1:**
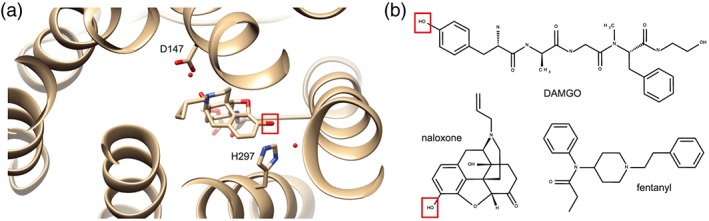
Structural concepts for the investigation of ligand‐specific pH‐sensitivity of binding to MOR. (a) Structure of the human μ‐opioid receptor bound to the antagonist β‐funaltrexamine (β‐FNA) modified from the PDB entry 4DKL (Manglik et al., 2012) with the software USCF Chimera (Pettersen et al., 2004). Histidine 297^6.52^ (H297^6.52^) forms a water‐mediated hydrogen bond to the hydroxyl group at C27 (red box) of β‐FNA. (b) Skeletal formulas of [D‐Ala2,N‐Me‐Phe4,Gly5‐ol]‐enkephalin (DAMGO), naloxone (NLX), and fentanyl; hydroxyl groups demonstrated (in case of DAMGO) or likely (in case of naloxone) to interact with μ‐opioid receptor residue H297^6.52^ are highlighted by red boxes

**T**he aim of this study was to examine pH dependency of binding and signalling in HEK cells transfected with wild‐type (WT) and mutated μ‐opioid receptors . We focused on the amino acid histidine 297^6.52^ (H297^6.52^, superscript indicates the Ballesteros–Weinstein numbering; Ballesteros & Weinstein, 1995), which is critically involved in μ‐opioid receptor ligand binding (Huang et al., 2015; Koehl et al., 2018; Manglik et al., 2012). This residue is topologically conserved across species (including humans) and class A GPCRs, and homologous histidines convey proton sensing to several proton‐activated receptors (Liu et al., 2010; Ludwig et al., 2003). We hypothesized that pH‐dependent protonation of H297^6.52^ in μ‐opioid receptors leads to alterations in the hydrogen bond network between receptor and ligand and, consequently, in binding and downstream signalling. By exchanging H297^6.52^ for alanine, we created a mutant μ‐opioid receptor (MOR‐H297^6.52^A). Alanine side chains are non‐ionizable and cannot form hydrogen bonds, irrespective of pH (Achilonu, Fanucchi, Cross, Fernandes, & Dirr, 2012; J. M. Berg, Gatto, Stryer, & Tymoczko, 2003). To assess ligand binding potential, we used https://www.guidetopharmacology.org/GRAC/LigandDisplayForward?ligandId=1638, a μ‐opioid receptor antagonist that is likely to form a hydrogen bond network with H297^6.52^ based on its structural similarity to https://www.guidetopharmacology.org/GRAC/LigandDisplayForward?ligandId=1631 (Manglik et al., 2012), https://www.guidetopharmacology.org/GRAC/LigandDisplayForward?ligandId=1647, an μ‐opioid receptor agonist that was shown to form hydrogen bonds with H297^6.52^ (Koehl et al., 2018), and https://www.guidetopharmacology.org/GRAC/LigandDisplayForward?ligandId=1626, a highly selective μ‐opioid receptor agonist. The latter does not include a hydroxyl group (Figure 1), an important interaction partner of H297^6.52^ via hydrogen bonds (Dosen‐Micovic, Ivanovic, & Micovic, 2006; Manglik et al., 2016). The comparison of these three ligands enabled us to examine mechanistic and structural details determining receptor binding of conventional opioids at acidic pH.

## 2 METHODS

### 2.1 Constructs and mutagenesis

The plasmid containing the cDNA encoding the FLAG‐epitope‐tagged rat μ‐opioid receptor (oprm1, NM_013071.2) in pcDNA™3.1 vector with geneticin resistance gene was provided by Prof. Christian Zöllner (University Hamburg, Germany) and was used to generate the mutant MOR‐H297^6.52^A. Primers for site‐directed mutagenesis were designed with Stratagene QuikChange Primer Design online software (Agilent Technologies, Waldbronn, Germany). Mutagenesis PCR and subsequent digestion of parental DNA was conducted using the QuickChange II XL Site‐Directed Mutagenesis Kit (Agilent Technologies). The resulting DNA was amplified in XL 10‐Gold Ultracompetent cells and isolated using QIAprep Spin Miniprep or QIAfilter Plasmid Maxi Kits (QIAGEN, Hilden, Germany). The presence of the mutation was verified by sequencing.

### 2.2 Cell cultures and transfection

HEK 293 cells (DSMZ—German Collection of Microorganisms and Cell Cultures, Braunschweig, Germany; RRID:CVCL_0045) were maintained in DMEM supplemented with FBS (Biochrom; Berlin Germany), penicillin (100 U·ml^−1^; Biochrom), and streptomycin (100 μg·ml^−1^; Biochrom), with or without geneticin (G418, 100 μg·ml^−1^; Biochrom), in 5% CO_2_ at 37°C. Cells were passaged 1:3–1:20 every second to third day from P8 to P28 depending on confluence; 24 hr after seeding, confluent HEK WT cells (70–90%) were incubated with 1 μg per 200‐μl transfection mix of each plasmid containing the different cDNAs using X‐treme GENE HP DNA (Roche, Mannheim, Germany) reagent following the supplier's recommendations. For stable transfection, pcDNA™3.1 carrying the cDNA of MOR‐H297^6.52^A was linearized with restriction enzyme BglII (NEB, Frankfurt am Main, Germany). After 48 hr, the medium containing the transfection reagent was removed and geneticin (G418; 100 μg·ml^−1^) was added with standard culture medium for selection of successfully transfected cells. Membrane expression of the transfected receptors was qualitatively verified via immunocytochemistry (data not shown). Stably transfected, polyclonal cell lines were cultured for a maximum of 23 passages.

### 2.3 Membrane preparation for radioligand and [^35^S]‐GTPγS binding

HEK cells, either untransfected, stably expressing MOR‐WT (HEK MOR‐WT), or MOR‐H297^6.52^A (HEK MOR‐H297^6.52^A), were grown in 175‐cm^2^ tissue culture flasks to approximately 90% confluence, then rinsed and harvested in Tris buffer (50 mM, Trizma preset crystals pH 7.4; Sigma‐Aldrich, Darmstadt, Germany). Cell suspensions were homogenized using a mechanical disperser (Dispergierstation T8.10, IKA‐Werke GmbH Co. KG, Staufen, Germany) at maximum speed for 10 s and centrifuged at 42,000 *g* and 4°C for 20 min (Avanti JXN‐26 ultracentrifuge, Beckmann Coulter, Krefeld, Germany). Supernatants were discarded, sediments were resuspended, homogenized, and centrifuged as above, and pellets were stored at −80°C. On the day of usage, pellets were thawed in Trizma buffer and homogenized, and protein concentration was determined in duplicates according to the Bradford method (Bio‐Rad Protein Assay Dye Reagent Concentrate, Bio‐Rad Laboratories GmbH, München, Germany). Homogenates were split according to the number of conditions tested and centrifuged, supernatants were discarded, and pellets were resuspended in the respective assay buffer.

### 2.4 Radioligand saturation binding

[^3^H]‐naloxone and [^3^H]‐[D‐Ala2,N‐Me‐Phe4,Gly5‐ol]‐enkephalin ([^3^H]‐DAMGO) saturation binding to membrane fractions of HEK MOR‐WT or HEK MOR‐H297^6.52^A was measured to delineate *K*_D_ and *B*_max_. The experiments on MOR‐WT used 120‐min incubation in HEM buffer (8‐mM HEPES, 8‐mM 4‐(2‐hydroxyethyl)‐1‐piperazinepropanesulfonic acid [EPPS], and 8‐mM 2‐(N‐morpholino)ethanesulfonic acid [MES]) at pH 7.4, 6.5, and 6.0 respectively. Experiments on MOR‐H297^6.52^A used 120‐min incubation in 50‐mM Tris buffer at pH 7.4. [^3^H]‐naloxone was serially diluted to yield 10 times concentrated working solutions. To determine non‐specific binding, membrane fractions at equivalents of 100‐μg protein were incubated in duplicates with [^3^H]‐labelled ligand for 120 min at room temperature at the respective pH values, in the presence or absence of unlabelled naloxone (10 μM). Free ligands were separated from the membrane fraction by rapid vacuum filtration through Whatman GF/B glass fibre filters soaked in Trizma buffer and polyethylenimine (0.1% *w*/*v*), followed by six washes with cold HEM buffer at the respective pH. After overnight incubation of filters in scintillation fluid Optiphase HISAFE 3 (Perkin Elmer, 1200‐437, Waltham, USA), bound radioactivity was measured by liquid scintillation spectrometry at 69% counting efficiency with a Wallac 1414 Win Spectral Liquid Scintillation Counter (Perkin Elmer). Specific binding, expressed in counts per minute (cpm), was calculated by subtracting unspecific binding and transformed into fmol of bound radioligand per mg of total protein to enable comparison with previously published data. Data were fit with non‐linear regression One site—specific binding. For comparison to previous studies (Selley et al., 1993), we also used a preincubation paradigm: HEK MOR‐WT were incubated in extracellular solution buffer at pH 7.4, 6.5, or 5.5 at 37°C and 5% CO_2_ for 20 min prior to preparation of membrane fractions. [^3^H]‐DAMGO binding and wash steps were performed in Tris buffer at pH 7.4 as described above (data supplied as supporting information only). Pilot experiments with radiolabelled fentanyl showed very high non‐specific binding and were therefore discontinued.

### 2.5 [^35^S]‐GTPγS binding

Guanosine‐5‐*O*‐(3‐[^35^S]thio)‐triphosphate ([^35^S]‐https://www.guidetopharmacology.org/GRAC/LigandDisplayForward?ligandId=4207) binding to membrane fractions of untransfected or transfected HEK cells was measured to quantify G‐protein activation. Membrane fractions were prepared as described above. Where stated, *Pertussis* toxin (PTX; 100 ng·ml^−1^) and/or cholera toxin (CTX; 500 ng·ml^−1^) were added to the cell culture medium and incubated overnight (16 hr) before preparation of membrane fractions. In analogy to (Ludwig et al., 2003), 50 μg of membrane fractions in duplicates were incubated with GDP (30 μM) and/or [^35^S]‐GTPγS (0.05 nM) for 120 min at 30°C in HEM G‐protein buffer (8‐mM HEPES, 8‐mM EPPS, 8‐mM MES, 100‐mM NaCl, 5‐mM MgCl_2_, 0.2‐mM EGTA, 1% BSA, 1‐mM DTT) at the indicated pH values. Basal [^35^S]‐GTPγS binding was assessed in the absence of μ‐opioid receptor ligand. Non‐specific [^35^S]‐GTPγS binding was assessed by adding unlabelled GTPγS (10 μM) in the absence of opioid ligands. Fentanyl (0.1 pM–100 μM), DAMGO (0.1 pM–100 μM), or naloxone (1–100 μM) were applied where stated. Free labelled nucleotides were separated from the membrane fraction by rapid vacuum filtration through Whatman GF/B glass fibre filters, followed by six washes with ice‐cold Trizma buffer. After overnight incubation of filters in scintillation fluid Optiphase HISAFE 3 (Perkin Elmer), bound radioactivity was measured by liquid scintillation spectrometry at 69% counting efficiency with a Wallac 1414 Win Spectral Liquid Scintillation Counter (Perkin Elmer), yielding cpm. Unspecific [^35^S]‐GTPγS binding was subtracted from the raw data to yield specific binding. In dose–response experiments, data were normalized to baseline binding (defined as 100%), and binding curves were fit with non‐linear regression log (agonist) versus response (three parameters) to obtain the EC_50_ and *E*_max_ (top of the curve) as measures of agonist potency and intrinsic efficacy respectively.

### 2.6 cAMP‐enzyme immunoassay

Cells were seeded into poly‐L‐lysine‐coated 96‐well plates the day prior to the experiment to yield 90–100% confluence. Where stated, PTX (100 ng·ml^−1^) was added to the cell culture medium and incubated overnight (16 hr) before experiments. At each ligand concentration and pH value, measurements were performed in duplicates or triplicates. On the day of the experiment, the medium was removed, and 100 μl of one of the following test solutions were added immediately: To optimize detection of opioid effects, cotreatment with IBMX (2 mM; to prevent https://www.guidetopharmacology.org/GRAC/LigandDisplayForward?ligandId=2352 degradation) and PGE_2_ (1 μM) or forskolin (10 μM; K. A. Berg et al., 2007; to stimulate cAMP production) were included in every well as indicated. These agents were diluted in extracellular solution (140‐mM NaCl, 5‐mM KCl, 2‐mM MgCl_2_, 2‐mM CaCl_2_, 10‐mM HEPES, and 10‐mM D‐(+)‐glucose) at pH 7.4, 6.5, and 6.0. Fentanyl was added at various doses (1 nM to 50 μM), or a fixed dose of fentanyl (10 μM) was combined with increasing doses of naloxone (1 nM to 1 mM). After 20‐min incubation at room temperature, cells were lysed, and intracellular cAMP levels were detected using Amersham cAMP Biotrak Enzymeimmunoassay kit (GE Healthcare, Solingen, Germany). Once lysed, all samples were handled at pH 7.4, following the manufacturers' recommendations. cAMP levels were quantified at 450 nm with a spectrophotometer plate reader (Spectra Max 340PC, Molecular Devices, Sunnyvale, USA). Data were normalized by setting PGE_2_‐ or forskolin‐stimulated cAMP levels in the absence of opioid ligands at the respective pH as 100% and a value of 0 as 0% to adjust for differences in baselines. Naloxone dose–response curves were fit with non‐linear regression log (agonist) versus response (three parameters), and fentanyl dose–response curves were fit with non‐linear regression log (antagonist) versus response (three parameters).

### 2.7 Data handling and statistical analysis

The data and statistical analysis comply with the recommendations of the *British Journal of Pharmacology* on experimental design and analysis in pharmacology (Curtis et al., 2018). We used randomized block design to control for position effects on plates (cAMP assay) or filter apparatus (radioligand and [^35^S]‐GTPγS binding). Data recording and analysis were not blinded due to obvious effects of pH on assay baselines. We performed six independent replicates of each experiment (*n* = 6). In cAMP enzyme immunoassays, single raw values were excluded from the triplicates if normalized values deviated from the overall mean by more than 2 SDs. This procedure resulted in exclusion of 11 raw values in fentanyl and 25 raw values in naloxone dose–response curves. Per data point (pH and drug concentration), no more than 2 raw values out of 18 (six experiments measured in triplicates) were excluded. GraphPad Prism5 (GraphPad, San Diego, USA) was used for all curve fittings, statistical analyses, and data graph generation, with one exception: GraphPad Prism 8.1.0 was used to perform one‐way ANOVA with Welch's correction for unequal SD, followed by Dunnett's T3 multiple comparison post hoc test. *P* values ≤.05 were considered significant. Normality of data was assessed using the Kolmogorov–Smirnov test. Data are represented as mean ± SEM (normally distributed data) or median with interquartile range (nonnormally distributed data). In dose–response experiments, concentrations were transformed to log scale, so data could be fit to sigmoidal dose–response curves. The pharmacological parameters and SEM derived from the fit curves were compared by one‐way ANOVA or one‐way ANOVA with Welch's correction if the SD varied significantly. Other data were compared by either one‐way or two‐way ANOVA. Dunnett's multiple comparison or Bonferroni post hoc tests were used to compare all conditions to MOR‐WT at pH 7.4, if the preceding one‐ or two‐way ANOVA revealed a significant difference. Dunnett's T3 multiple comparison post hoc test was used following one‐way ANOVA with Welch's correction. Homogeneity of sample variances was assessed via Bartlett's test for equal variances. Non‐normally distributed data were analysed by a Kruskal–Wallis test, followed by Dunn's post hoc test if initial *p* values were ≤ .05. Molecular graphics were created with UCSF Chimera, developed by the Resource for Biocomputing, Visualization, and Informatics at the University of California, San Francisco (Pettersen et al., 2004).

### 2.8 Materials

All chemicals were purchased from Sigma‐Aldrich (Darmstadt, Germany), unless stated otherwise. [^3^H]‐naloxone, [^3^H]‐DAMGO, and [^35^S]‐GTPγS were purchased from Perkin Elmer (Waltham, USA). FBS, penicillin/streptomycin, and geneticin (G418) were purchased from Biochrom (Berlin, Germany).

### 2.9 Nomenclature of targets and ligands

Key protein targets and ligands in this article are hyperlinked to corresponding entries in http://www.guidetopharmacology.org, the common portal for data from the IUPHAR/BPS Guide to PHARMACOLOGY (Harding et al., 2018), and are permanently archived in the Concise Guide to PHARMACOLOGY 2017/18 (Alexander et al., 2017).

## 3 RESULTS

### 3.1 Influence of pH and H297^6.52^A on μ‐opioid receptor ligand binding

**Figure 2:**
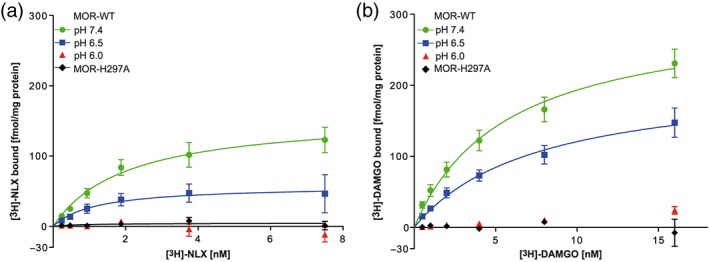
Dependence of naloxone and DAMGO binding on pH and H297^6.52^. (a) [^3^H]‐naloxone ([^3^H]‐NLX) and (b) [^3^H]‐DAMGO saturation binding to MOR‐WT and MOR‐H297^6.52^A. Data represent mean ± SEM of fmol of bound [^3^H]‐naloxone or [^3^H]‐DAMGO per mg of total protein with non‐linear fit. Derived parameters are shown in Table 1; *B*_max_ values for naloxone are significantly different (*P* < .05) between pH 6.5 and 7.4; *n* = 6 per experiment

We have previously demonstrated that fentanyl binding to μ‐opioid receptors is not significantly altered at low pH (Rodriguez‐Gaztelumendi, Spahn, Labuz, Machelska, & Stein, 2018; Spahn et al., 2017; Spahn et al., 2018). In this study, we examined the binding of naloxone, which, in contrast to fentanyl, is likely to form hydrogen bonds with H297^6.52^ (Figure 1). [^3^H]‐naloxone saturation binding experiments with MOR‐WT showed progressively decreasing binding with declining pH values (Figure 2a). The *B*_max_ at pH 6.5 was significantly reduced compared to pH 7.4. Interestingly, the *K*_D_ of [^3^H]‐naloxone was not influenced by pH variation. At pH 6.0, we found no specific [^3^H]‐naloxone binding (Figure 2a). In MOR‐H297^6.52^A, we found almost no specific [^3^H]‐naloxone binding at pH 7.4 (Figure 2a, Table 1). MOR‐H297^6.52^A was investigated only at pH 7.4 because alanine side chains cannot be protonated (J. M. Berg et al., 2003).

**Table 1:** [^3^H]‐naloxone and [^3^H]‐DAMGO binding

Ligand	Parameter	MOR‐WT pH 7.4	MOR‐WT pH 6.5	MOR‐WT pH 6.0	MOR‐H297^6.52^A pH 7.4
[^3^H]‐naloxone	*K*_D_ (nM)	2.0 ± 0.7	1.2 ± 1.1	No fit	0.8 ± 2.2
	*B*_max_ (fmol·mg^−1^ protein)	158.3 ± 22.0	57.8 ± 17.1*	No fit	4.6 ± 3.7*
[^3^H]‐DAMGO	*K*_D_ (nM)	5.7 ± 1.4	7.5 ± 2.5	No fit	No fit
	*B*_max_ (fmol·mg^−1^ protein)	304.1 ± 32.6	211.5 ± 33.6	No fit	No fit

*Note*. Data are means ± SEM, *n* = 6 per experiment.

* *P* < .05, *K*_D_ and *B*_max_ were each compared by unpaired *t* test.

Abbreviations: MOR‐WT, μ‐opioid receptor wild type; MOR‐H297^6.52^A, μ‐opioid receptor mutated at H 297^6.52^ to A.

To examine whether this effect also occurs with agonists, we used [^3^H]‐DAMGO, which can form hydrogen bonds with H297^6.52^ (Koehl et al., 2018). At pH 6.5, a trend towards a decrease in maximum [^3^H]‐DAMGO binding was observed. At pH 6.0, [^3^H]‐DAMGO binding was abolished. In addition, [^3^H]‐DAMGO binding was absent on MOR‐H297^6.52^A (Figure 2b, Table 1). Preincubation of membrane fractions at low or physiological pH for 20 min, as in Selley et al. (1993), did not alter [^3^H]‐DAMGO binding after returning to physiological pH (Figure S1).

### 3.2 Influence of low pH and H297^6.52^A on [^35^S]‐GTPγS binding

**Figure 3:**
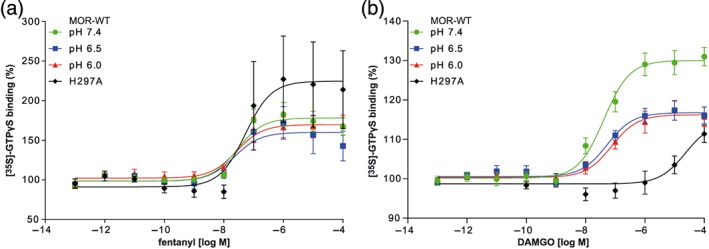
Dependence of [^35^S]‐GTPγS binding on pH and H297^6.52^. (a) Fentanyl‐ and (b) DAMGO‐induced [^35^S]‐GTPγS binding to MOR‐WT and in MOR‐H297^6.52^A. Data represent mean ± SEM of specific [^35^S]‐GTPγS binding in % of baseline with non‐linear fit. *n* = 6 per curve. Derived parameters are shown in Table 2. (a) No significant differences were found; (b) significant differences (*P* < .05) from MOR‐WT pH 7.4 were found for MOR‐WT at pH 6.5 and pH 6.0 (*E*_max_) and MOR‐H297^6.52^A (logEC_50_ and *E*_max_)

Neither pH nor mutation of H297^6.52^ altered fentanyl‐induced [^35^S]‐GTPγS binding significantly (Figure 3a). However, shifts in baseline [^35^S]‐GTPγS binding must be considered (see below). As shown in Table 2, EC_50_ values and maximum intrinsic efficacy (*E*_max_) did not statistically differ across the pH conditions or receptor genotypes. In contrast, DAMGO‐induced [^35^S]‐GTPγS binding in MOR‐WT membranes was significantly reduced at pH 6.5 and 6.0 compared to pH 7.4. At MOR‐H297^6.52^A, the dose–response curve of DAMGO‐induced [^35^S]‐GTPγS binding was significantly shifted to the right (Table 2).

**Table 2:** Fentanyl‐ and DAMGO‐induced [^35^S]‐GTPγS binding

	Parameter	MOR‐WT	MOR‐WT	MOR‐WT	MOR‐H297^6.52^A
		pH 7.4	pH 6.5	pH 6.0	pH 7.4
Fentanyl	EC_50_ (nM) [95% CI] log (EC_50_)	28.0 [12.9, 59.8] −7.55 ± 0.22	22.5 [4.9, 90.8] −7.65 ± 0.42	26.3 [9.1, 75.9] −7.58 ± 0.27	52.8 [8.9, 328.8] −7.28 ± 0.47
	*E*_max_ (%)	178.1 ± 5.1	159.8 ± 7.2	169.6 ± 5.3	224.6 ± 19.9
DAMGO	EC_50_ (nM) [95% CI] log (EC_50_)	41.4 [20.7, 79.3] −7.38 ± 0.13	53.2 [23.2, 115.3] −7.27 ± 0.17	75.5 [27.4, 195.5] −7.12 ± 0.20	23 μM [5.7 μM, unknown] −4.63 ± 0.33*
	*E*_max_ (%)	130.0 ± 1.2	116.7 ± 0.9*	116.2 ± 1.1*	114.3 ± 3.5*

*Note*. Data are means ± SEM or means with 95% confidence interval (95% CI); *n* = 6 per experiment. log (EC_50_) values were compared by one‐way ANOVA with Dunnett's multiple comparison post hoc test, control group = MOR‐WT pH 7.4. E_max_ values were compared by one‐way ANOVA with Welch's correction for unequal SD, followed by Dunnett's T3 multiple comparison post hoc test. All comparisons were performed within agonist groups.

Abbreviations: *E*_max_, maximum intrinsic activity; MOR‐WT, wild type μ‐opioid receptor; MOR‐H297^6.52^A, μ‐opioid receptor mutated at H 297^6.52^ to A.

### 3.3 Influence of pH and H297^6.52^A on cAMP concentration

**Figure 4:**
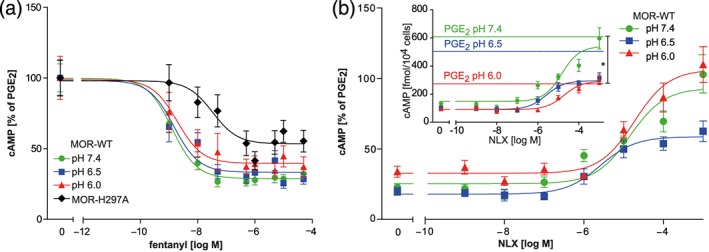
Dependence of cAMP modulation on pH and H297^6.52^. (a) Fentanyl‐induced cAMP reduction in HEK MOR‐WT and in HEK MOR‐H297^6.52^A. Data represent means ± SEM of cAMP accumulation in % of the values obtained with PGE_2_ alone at the respective pH, and non‐linear fit; *n* = 6 per curve. Derived parameters are shown in Table 3 (upper panel); significant differences (*P* < .05) between MOR‐WT pH 7.4 and MOR‐H297^6.52^A (logEC_50_ and Top). (b) Naloxone antagonism of fentanyl‐induced cAMP inhibition in HEK MOR‐WT. Data represent means ± SEM in % of PGE_2_‐induced baselines at the respective pH (inset: same data before normalization in fmol/10^4^ cells with PGE_2_‐stimulated baselines) with non‐linear fit; *n* = 6 per curve. Derived parameters are shown in Table 3 (lower panel), significant differences (*P* < .05) were found between pH 6.5 and pH 7.4 (logEC_50_ and Top)

The IC_50_ and maximum effect of fentanyl‐induced cAMP reduction in HEK MOR‐WT were not affected by pH (Table 3). In HEK MOR‐H297^6.52^A, fentanyl‐induced cAMP reduction was significantly less pronounced compared to MOR‐WT, suggesting a minor contribution of H297^6.52^ to activation of μ‐opioid receptors by fentanyl (Figure 4a, Table 3).

**Table 3:** Effects of fentanyl and naloxone on cAMP content

Effect	Parameter	MOR‐WT	MOR‐WT	MOR‐WT	MOR‐H297A
		pH 7.4	pH 6.5	pH 6.0	pH 7.4
Fentanyl‐induced cAMP reduction	IC_50_ (nM) [95% CI] log (IC_50_)	1.3 [0.7, 2.4] −8.90 ± 0.14	1.4 [0.5, 4.0] −8.71 ± 0.33	1.8 [0.4, 8.6] −8.74 ± 0.34	33.4 [5.6, 198.6] −7.48 ± 0.40*
	Bottom (%)	28.8 ± 2.1	33.2 ± 2.9	39.5 ± 4.0	53.3 ± 4.7*
Naloxone antagonism of fentanyl‐induced cAMP reduction	IC_50_ (μM) [95% CI] log (IC_50_)	14.4 [5.8, 35.9] −4.84 ± 0.20	2.6 [0.9, 7.7] −5.58 ± 0.25*	18.7 [7.6, 45.6] −4.73 ± 0.20	
	Top (%)	93.1 ± 5.7	58.7 ± 3.4*	106.3 ± 6.3	

*Note*. Data are means ± SEM or means with 95% CI. Bottom: bottom of curve fit (maximum fentanyl effect); Top: top of curve fit (maximum reversal of fentanyl effect); log (IC_50_), Bottom and Top were each compared by one‐way ANOVA with Dunnett's Multiple Comparison post hoc test, control group = MOR‐WT pH 7.4.

* *P* < 0.05, *n* = 6 per experiment.

We next assessed naloxone antagonism of fentanyl‐induced cAMP reduction. At pH 6.5, the maximum effect (see Top %) and the logIC_50_ of naloxone antagonism were significantly reduced compared to pH 7.4 (Table 3, Figure 4b). At pH 6.0 and the highest concentration of naloxone, the absolute cAMP content (fmol per 10^4^ cells; before normalization) was significantly decreased. As naloxone did not show binding to MOR‐H297^6.52^A (Figure 2a), we did not further test the effects of naloxone on fentanyl‐induced cAMP inhibition in this cell line. Again, shifts in baseline cAMP concentrations must be considered (see below).

### 3.4 Influence of low pH on baseline [^35^S]‐GTPγS binding

**Figure 5:**
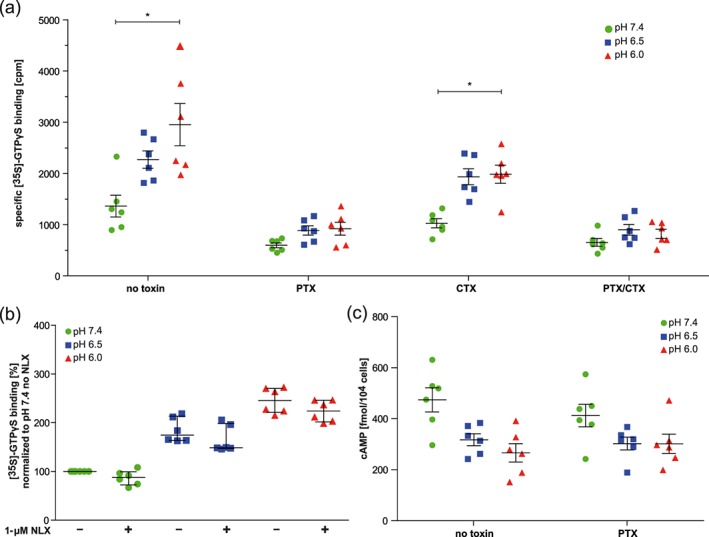
Effects of pH and toxins on basal [^35^S]‐GTPγS binding and cAMP contents without opioid ligands. (a) Basal [^35^S]‐GTPγS binding to MOR‐WT, pretreated (overnight) or not with *Pertussis* toxin (PTX), cholera toxin (CTX), or both (PTX/CTX). Data represent specific [^35^S]‐GTPγS binding in cpm by experiment (dots) with means ± SEM (line and bars); *n* = 6 per condition. *P < .05, significantly different as indicated; one‐way ANOVA with Welch's correction for unequal SD with Dunnett's T3 multiple comparison post hoc test (pH 7.4 vs. pH 6.0 within treatment groups). (b) Basal [^35^S]‐GTPγS binding to MOR‐WT, in the absence and presence of 1‐μM naloxone (NLX). Data represent specific [^35^S]‐GTPγS binding in cpm by experiment (dots) with median and interquartile range (lines and bars) of [^35^S]‐GTPγS bound in % of pH 7.4 (without naloxone). *n* = 6 per condition; Friedman test with Dunn's Multiple Comparison Test yielded no significant effects of naloxone treatment. (c) cAMP production in HEK MOR‐WT pretreated (overnight) or not with PTX (and then stimulated with PGE_2_). Data represent cAMP levels in fmol per 10^4^ cells by experiment (dots) with mean ± SEM (lines and bars), *n* = 6 per condition., *P* < .05, significant source of interaction for pH, not for treatment; two‐way ANOVA

Without opioid ligands, basal [^35^S]‐GTPγS binding increased with declining pH values in HEK MOR‐WT (Figure 5a, left panel without toxins). To examine whether this effect was due to activation of Gα_i_ or Gα_s_, we used toxins to block the respective subunits (Figure 5a). Blocking of Gα_i_ subunits alone (with PTX) or in combination with Gα_s_ (with PTX/CTX) abolished the pH‐dependent increase in basal [^35^S]‐GTPγS binding, while such increase persisted after blocking Gα_s_ alone (with CTX). Naloxone (1 μM) did not significantly alter basal [^35^S]‐GTPγS binding, irrespective of pH (Figure 5b). Notably, the pH‐dependent increase in [^35^S]‐GTPγS binding in MOR‐WT appeared to be comparable to that in untransfected HEK cells (Figure S4).

### 3.5 Influence of low pH on baseline cAMP concentration

Low pH decreased PGE_2_‐stimulated cAMP baselines (Figures S2 and 4b). In contrast to [^35^S]‐GTPγS binding, this pH effect on cAMP baselines persisted after treatment with PTX (Figure 5c). PTX activity was maintained at all pH values (Figure S4A). The cAMP content in the absence of PGE_2_ was not significantly different between low and physiological pH (Figure S4B).

## 4 DISCUSSION

In this study, we have investigated the role of pH and H297^6.52^ on ligand binding, G‐protein activation, and downstream signalling by μ‐opioid receptors. Our data suggest that low pH impairs binding and signalling selectively for ligands that can form hydrogen bond networks with the μ‐opioid receptor histidine residue, H297^6.52^. In particular, we demonstrated that the single mutation of H297^6.52^ to alanine was sufficient to decrease binding and subsequent modulation of intracellular signalling of pH sensitive μ‐opioid receptor ligands, regardless of their agonist or antagonist properties.

In our saturation binding experiments, low pH reduced the number of binding sites for [^3^H]‐naloxone, while the affinity was unaffected. Similarly, mutation of H297^6.52^ to alanine greatly reduced naloxone binding, suggesting that naloxone binding indeed depends on H297^6.52^. Based on a comparison of the structures of naloxone and β‐funaltrexamine , we assumed that naloxone is likely to form a hydrogen bond network involving the hydroxyl group at position C21 and H297^6.52^ of μ‐opioid receptors (Manglik et al., 2012). Naloxone is largely protonated at both physiological and acidic pH (Mazak, Hosztafi, & Noszal, 2015). Therefore, alterations in the pH 7.4 to 6.0 will not markedly alter the protonation state of naloxone. This suggests that effects of acidic pH on naloxone binding are likely to be due to protonation of the receptors. Importantly, both low pH and mutation of H297^6.52^ to A also resulted in impaired binding of the agonist [^3^H]‐DAMGO. DAMGO is similarly able to form a hydrogen bond network with H297^6.52^ (Koehl et al., 2018). Of note, 20‐min exposure to low pH prior to preparation of membrane fractions did not alter DAMGO binding after changing the pH back to 7.4, suggesting that the loss of DAMGO binding at pH 6.0 was not due to irreversible damage of μ‐opioid receptors by low pH.

Our binding experiments on MOR‐H297^6.52^A were performed in Tris buffer, because it is conventionally used to study μ‐opioid receptor radioligand binding, and MOR‐H297^6.52^A was investigated only at pH 7.4 because alanine side chains cannot be protonated. The binding of [^3^H]‐naloxone and [^3^H]‐DAMGO binding to MOR‐WT were investigated in HEM buffer to cover a wider pH range. Pilot experiments indicated that [^3^H]‐naloxone binding to MOR‐WT did not differ between Tris and HEM buffer at pH 7.4.

In contrast to naloxone and DAMGO, we previously showed that fentanyl binding to μ‐opioid receptors was not altered by different pH conditions (Rodriguez‐Gaztelumendi et al., 2018; Spahn et al., 2017). Our preliminary experiments with radiolabelled fentanyl showed excessive non‐specific binding, which precluded analysis of fentanyl binding to MOR‐H297^6.52^A. Simulations of fentanyl binding suggest that it does not form hydrogen bonds with H297^6.52^, while other polar interactions are maintained (Dosen‐Micovic et al., 2006). Altogether, our data suggest that low pH selectively impairs binding of ligands that can establish hydrogen bond networks with the WT residue H297^6.52^, in μ‐opioid receptors, but not of ligands that do not form such bonds. These findings confirm earlier studies investigating low pH effects on opioid binding (Pert & Snyder, 1973; Smith, 1977) and expand those in that we suggest a ligand‐specific mechanism.

To examine pH effects on G‐protein activation, we employed [^35^S]‐GTPγS binding assays and observed that basal (ligand‐independent) G‐protein activation was significantly enhanced at low pH. Baseline differences between groups usually hinder data interpretation (Curtis et al., 2015). Yet they appear frequently when studying pH effects (Hugel, Kadiri, Rodeau, Gaillard, & Schlichter, 2012; Ludwig et al., 2003; J. Q. Wang et al., 2004). Therefore, we investigated baseline effects to enable interpretation of ligand‐dependent data and increase transparency. Alterations in pH were previously reported to change baseline values of [^35^S]‐GTPγS binding and cAMP concentrations (J. Q. Wang et al., 2004), as well as thermodynamic stability of the Gα_i_‐GTPγS complex and GTPase activity of other GTP‐binding proteins (Isom et al., 2013; Mendieta et al., 2009). Our data suggest that the increased [^35^S]‐GTPγS binding at low pH was not caused by off‐target binding of GTP, as it was blocked by overnight preincubation with PTX, indicating that [^35^S]‐GTPγS was indeed bound to (inhibitory) G‐proteins. Furthermore, naloxone did not modulate basal [^35^S]‐GTPγS binding, irrespective of pH, suggesting that protons per se did not activate opioid receptors. Notably, naloxone may act as neutral antagonist or inverse agonist, depending on the context (Divin, Bradbury, Carroll, & Traynor, 2009; Masuho et al., 2015; D. Wang, Raehal, Bilsky, & Sadee, 2001). We performed [^35^S]‐GTPγS assays under conditions of low constitutive activity of the μ‐opioid receptors, which precluded the investigation of inverse agonism (Heusler, Tardif, & Cussac, 2016). If acidic pH had increased the constitutive activity of these receptors, we would have expected naloxone to counteract this at pH 6.5, as binding at this pH was maintained. In addition, the increased [^35^S]‐GTPγS baselines appeared comparable between untransfected and HEK MOR‐WT cells. We conclude that the increase in [^35^S]‐GTPγS baseline values at low pH is independent of μ‐opioid receptor expression and is likely to be due to pH‐dependent reduction of GTPγS hydrolysis, as suggested in previous studies (Isom et al., 2013; Ott & Costa, 1989; Selley et al., 1993). Thus, to enable data comparison between the different pH values, we normalized ligand‐dependent [^35^S]‐GTPγS binding to baselines.

We further demonstrated that fentanyl‐induced [^35^S]‐GTPγS binding was not altered by pH, providing evidence that not only fentanyl binding (as shown in our previous studies) but also fentanyl‐induced signalling is independent of pH changes towards a more acidic milieu. Likewise, fentanyl‐induced [^35^S]‐GTPγS binding was not altered in MOR‐H297^6.52^A compared to MOR‐WT, indicating that activation of μ‐opioid receptors by fentanyl does not depend on H297^6.52^. In contrast, DAMGO‐induced [^35^S]‐GTPγS binding was reduced at acidic pH. In membranes of MOR‐H297^6.52^A expressing cells, DAMGO‐induced G‐protein activation was nearly abolished. Both observations corroborate our findings of pH‐ and H297^6.52^‐dependent DAMGO binding.

Finally, we investigated downstream effects of acidic pH on modulation of cAMP production by fentanyl and naloxone. In line with the increased basal Gα_i_ activity in [^35^S]‐GTPγS binding experiments, decreasing extracellular pH progressively reduced the cAMP‐accumulation stimulated by PGE_2_. In pilot experiments, we observed similarly reduced cAMP levels at low pH after treatment with forskolin. The pH‐dependent decrease in cAMP‐accumulation might reflect the enhanced basal activity of inhibitory G‐proteins. However, in contrast to G‐protein activation, PTX did not inhibit this effect. This suggests that the observed reduction of PGE_2_‐stimulated cAMP production at pH 6.0 was not mediated by activation of Gα_i_. Without direct stimulants of AC or opioids, cAMP levels did not significantly differ between pH 7.4 and 6.0. Taken together, we attribute the reduced cAMP levels at pH 6.0 to the previously demonstrated pH‐dependence of AC activity (Ludwig et al., 2003; J. Q. Wang et al., 2004). Due to the significant baseline reduction at pH 6.0, we only considered data obtained at pH 6.5 and 7.4 for analysis.

When examining the modulation of cAMP levels by μ‐opioid receptor ligands, we found that fentanyl‐induced reduction of cAMP concentration was independent of pH but somewhat impaired in MOR‐H297^6.52^A. These data suggest that fentanyl‐induced μ‐opioid receptor signalling is not entirely independent of the residue H297^6.52^ but does not interact with this residue via hydrogen bonds (Dosen‐Micovic et al., 2006). Instead, H297^6.52^ might stabilize fentanyl binding via van der Waals or π‐stacking interactions. In addition, altered receptor expression patterns in MOR‐H297^6.52^A cells cannot be ruled out since we did not quantify MOR‐WT and MOR‐H297^6.52^A expression levels. However, our finding that fentanyl elicited strong dose‐dependent [^35^S]‐GTPγS binding in both MOR‐WT and MOR‐H297^6.52^A membranes (Figure 3) suggests that cell surface amounts of WT and mutant receptors were similar. Endogenous expression of MOR‐WT in HEK 293 cells at sufficiently high levels to account for the fentanyl‐induced cAMP response in MOR‐H297^6.52^A cells is unlikely, because endogenous receptors should have elicited [^35^S]‐GTPγS binding in response to DAMGO, which we did not find (Figure 3). We conclude that fentanyl modulates cAMP levels also via MOR‐H297^6.52^A.

In line with reduced naloxone binding at low pH, naloxone antagonism of fentanyl‐induced cAMP reduction was impaired at pH 6.5 compared to pH 7.4. Because naloxone did not bind with high affinity at low pH, only high concentrations of naloxone (10 μM or higher) significantly reversed fentanyl effects in cAMP assays at pH 6.0. Unexpectedly, the naloxone dose–response at pH 7.4 displayed an irregular, tendentially biphasic shape. We assume that this was caused by the high fentanyl concentration used in these competition experiments. Nevertheless, the selective inhibitory effect of acidic pH on naloxone binding clearly translated into downstream signalling.

In summary, our data suggest that protonation of H297^6.52^ reduces naloxone and DAMGO affinity to μ‐opioid receptor below the measurable range in saturation binding, resulting in a reduction, but not abolition, of effects on agonist‐induced downstream signalling. In contrast, fentanyl binding and downstream signalling were found to be independent of pH and, therefore, possibly of the protonation state of H297^6.52^. Given the striking structural differences between the morphinan‐based antagonist naloxone and the peptide agonist DAMGO, we suggest that the observed influence of low pH on binding and signalling can be generalized to other opioid ligands that are capable of forming hydrogen bonds to H297^6.52^. Unfortunately, naloxone and DAMGO did not retain enough functionality at MOR‐H297^6.52^A to assess whether a pH effect persisted in the absence of H297^6.52^. It is clear that pH‐dependent ligand binding is not the sole role of H297^6.52^ (see, e.g., Toll, Bruchas, Calo, Cox, & Zaveri, 2016). We did not test replacement of H297^6.52^ by acidic or polar uncharged residues because the possible functional implications are beyond the scope of the current study (Spivak et al., 1997). Meanwhile, histidines often mediate pH‐dependent changes of proteins in the pathological range (Valery et al., 2015). The pK_a_ of histidine in folded proteins lies in the range of 6.6 ± 1.0 (Grimsley, Scholtz, & Pace, 2009). Therefore, inflammation‐induced changes in pH (see Stein, 2018) can conceivably alter the protonation state of histidine side chains. At physiological pH, these residues are in the monoprotonated neutral form, whereas at low pH, both imidazole nitrogens of histidine are likely to be protonated, thereby changing the nature of the side chain from potential H‐bond acceptor to potential H‐bond donor (see, for example, Li & Hong, 2011). An early study by Smith (1977) anticipated that pH‐dependent opioid binding was mediated by an anionic group with a pK_a_ of 7.0 within the receptor. According to the Henderson–Hasselbalch equation, a pK_a_ of 7.0 would yield 71.4% deprotonated H297^6.52^ at pH 7.4, and 24.2% deprotonated H297^6.52^ at pH 6.5. These proportions match our observations in that the *B*_max_ of naloxone at pH 7.4 was roughly three times that at pH 6.5.

We conclude that protonation of the highly conserved H297^6.52^ in theμ‐opioid receptor binding pocket ligand specifically reduces binding and downstream signalling. Expanding these investigations to other opioid ligands might complement structural studies, which were so far limited to physiological conditions. For the first time, we have now demonstrated structural requirements for optimal opioid ligand recognition in acidic environments. Our data enhance understanding of the effects of low pH on μ‐opioid receptor‐ligand interactions, which is essential for structure‐based drug design.

## AUTHOR CONTRIBUTIONS

J.M., G.D.V., V.S., and N.M. designed and conducted the experiments. J.M., G.D.V., and C.S. analysed the data. J.M., G.D.V., V.S., N.M. and C.S. wrote the manuscript.

## CONFLICT OF INTEREST

The authors declare no conflicts of interest.

## DECLARATION OF TRANSPARENCY AND SCIENTIFIC RIGOUR

This Declaration acknowledges that this paper adheres to the principles for transparent reporting and scientific rigour of preclinical research as stated in the *BJP* guidelines for https://bpspubs.onlinelibrary.wiley.com/doi/abs/10.1111/bph.14207, and as recommended by funding agencies, publishers and other organisations engaged with supporting research.

## Supporting information

Figure S1.
[^3^H]‐DAMGO binding after pre‐incubation at low pH.
Figure S2. Fentanyl effects on cAMP content in HEK MOR‐WT and MOR‐H297^6.52^A.
Figure S3. Basal [^35^S]‐GTPγS binding at physiological and low pH.
Figure S4. Effects of pertussis toxin on cAMP accumulation and basal cAMP concentrations in intact HEK MOR‐WT cells at different pH values.
