# Supplementary material for: Modulation of μ‐opioid receptor activation by acidic pH is dependent on ligand structure and an ionizable amino acid residue
Source: Br J Pharmacol. 2019 Dec 5;176(23):4510–20. doi: 10.1111/bph.14810 (PMC6932940; doi:10.1111/bph.14810)
Supplement: Supplementary file 1 — Figure S1. [3H]‐DAMGO binding after pre‐incubation at low pH. Figure S2. Fentanyl effects on cAMP content in HEK MOR‐WT and MOR‐H2976.52A. Figure S3. Basal [35S]‐GTPγS binding at physiological and low pH. Figure S4. Effects of pertussis toxin on cAMP accumulation and basal cAMP concentrations in intact HEK MOR‐WT cells at different pH values. [file BPH-176-4510-s001.pdf]

Supporting Information to manuscript "Modulation of mu-opioid receptor activation by acidic pH is dependent on ligand structure and an ionizable amino acid residue"

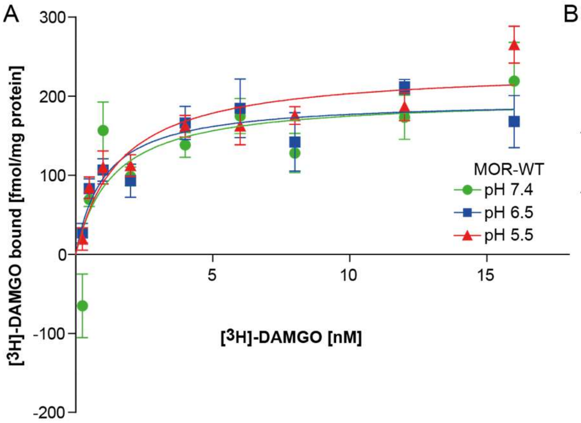

B

Parameter	MOR-WT pH 7.4	MOR-WT pH 6.5	MOR-WT pH 5.5
Kd [nM]	1.4 ± 0.7	1.0 ± 0.4	1.6 ± 0.4
Bmax [fmol / mg protein]	198.6 ± 25.6	195.5 ± 16.9	235.8 ± 17.4

Supporting Figure S1. [^{3}H]-DAMGO binding after pre-incubation at low pH

[^{3}H]-DAMGO saturation binding to membrane fractions of HEK MOR-WT at pH 7.4, following pre-incubation for 20 min at different pH values. Data represent means ± SEM of fmol of bound [^{3}H]-DAMGO per mg of total protein with nonlinear fit. (**B**) Parameters derived from curve fits in A. K_d and B_{max} were each compared by One-way ANOVA, no significant differences were found, *n* = 6 per experiment.

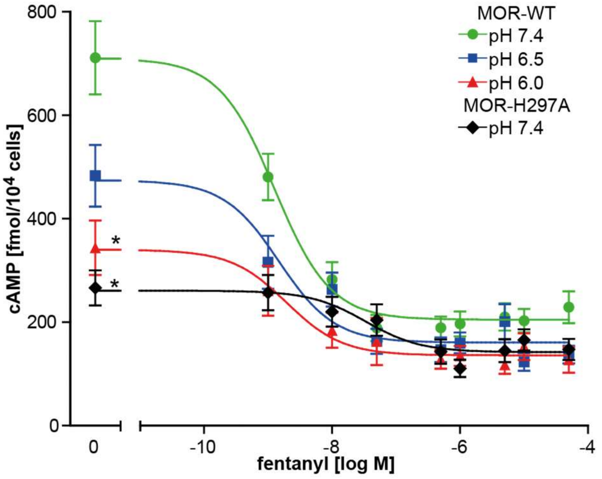

Supporting Figure S2. Fentanyl effects on cAMP content in HEK MOR-WT and MOR-H297$^{6.52}$A.

Fentanyl-induced cAMP reduction in HEK MOR-WT and HEK MOR-H297$^{6.52}$A. Data are expressed as cAMP per 10^4 cells and represent mean ± SEM with nonlinear fit. Baselines in absence of fentanyl were analyzed by One-way ANOVA with Dunnett's Multiple Comparison Test (control group = MOR-WT pH 7.4), * $P < 0.05$, n= 6 per curve.

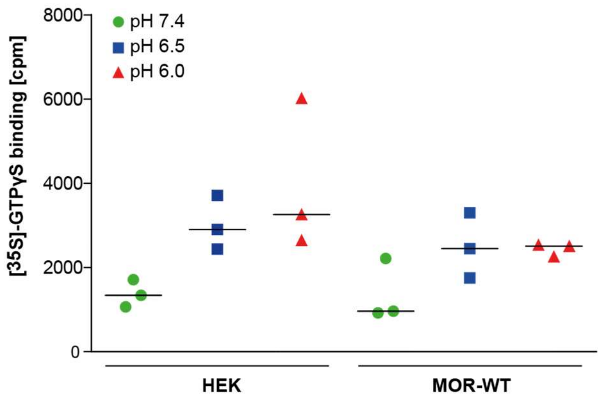

Supporting Figure S3. Basal [^{35}S]-GTPγS binding at physiological and low pH

Basal [^{35}S]-GTPγS binding at pH 7.4, 6.5 and 6.0 in membrane fractions of HEK WT and HEK MOR-WT in absence of opioid ligands. Data represent specific [^{35}S]-GTPγS bound in cpm by experiment (dots) with mean (lines), *n* = 3 per condition.

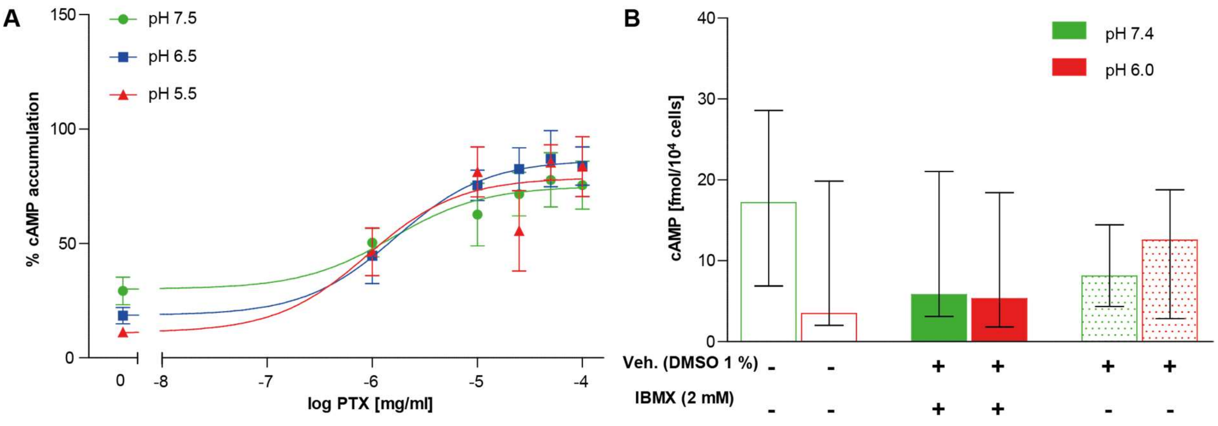

Supporting Figure S4. Effects of pertussis toxin on cAMP accumulation and basal cAMP concentrations in intact HEK MOR-WT cells at different pH values

(**A**) Reversal of fentanyl-induced cAMP reduction by PTX in HEK MOR-WT cells. Data are normalized to cAMP levels in absence of fentanyl and PTX at the respective pH and represent mean ± SEM with nonlinear fit. Comparison of fits with extra sum of squares F test (Top, $logEC_{50}$), ns, one curve for all datasets, n = 5 per curve. (**B**) cAMP accumulation in HEK MOR-WT at extracellular pH 7.4 or 6.0, in absence or presence of dimethyl sulfoxide (DMSO) and/or 3-isobutyl-1-methylxanthine (IBMX). Data represent median with interquartile range of cAMP in fmol/ 10^4 cells, Friedman test, ns, n = 6 per condition.
